# Fast and Simple Detection of *Yersinia pestis* Applicable to Field Investigation of Plague Foci

**DOI:** 10.1371/journal.pone.0054947

**Published:** 2013-01-29

**Authors:** Stéphanie Simon, Christian Demeure, Patricia Lamourette, Sofia Filali, Marc Plaisance, Christophe Créminon, Hervé Volland, Elisabeth Carniel

**Affiliations:** 1 CEA Saclay, iBiTec-S, Service de Pharmacologie et d’Immunoanalyse, Laboratoire d’Etudes et de Recherches en Immunoanalyse, Gif sur Yvette, France; 2 Institut Pasteur, Yersinia Research Unit and WHO Collaborating Center for Yersinia, Paris, France; Universidad de Costa Rica, Costa Rica

## Abstract

*Yersinia pestis*, the plague bacillus, has a rodent-flea-rodent life cycle but can also persist in the environment for various periods of time. There is now a convenient and effective test (F1-dipstick) for the rapid identification of *Y. pestis* from human patient or rodent samples, but this test cannot be applied to environmental or flea materials because the F1 capsule is mostly produced at 37°C. The plasminogen activator (PLA), a key virulence factor encoded by a *Y. pestis*-specific plasmid, is synthesized both at 20°C and 37°C, making it a good candidate antigen for environmental detection of *Y. pestis* by immunological methods. A recombinant PLA protein from *Y. pestis* synthesized by an *Escherichia coli* strain was used to produce monoclonal antibodies (mAbs). PLA-specific mAbs devoid of cross-reactions with other homologous proteins were further cloned. A pair of mAbs was selected based on its specificity, sensitivity, comprehensiveness, and ability to react with *Y. pestis* strains grown at different temperatures. These antibodies were used to develop a highly sensitive one-step PLA-enzyme immunoassay (PLA-EIA) and an immunostrip (PLA-dipstick), usable as a rapid test under field conditions. These two PLA-immunometric tests could be valuable, in addition to the F1-disptick, to confirm human plague diagnosis in non-endemic areas (WHO standard case definition). They have the supplementary advantage of allowing a rapid and easy detection of *Y. pestis* in environmental and flea samples, and would therefore be of great value for surveillance and epidemiological investigations of plague foci. Finally, they will be able to detect natural or genetically engineered F1-negative *Y. pestis* strains in human patients and environmental samples.

## Introduction

Despite public health measures implemented to eradicate plague, the disease persists in several countries, and is even reemerging [1]. Plague persistence is partly explained by the fact that it is a zoonotic disease with rodents as reservoirs and fleas as vectors. *Yersinia pestis* maintains itself among a population of partially resistant rodents and, when transmitted to more susceptible animals, leads to epizootics [2].

Although *Y. pestis* is commonly believed to be unable to survive outside a flea or a mammalian host [3], different works documented the concept of burrowing plague by showing that *Y. pestis* could survive for several years in the burrows of dead rodents [4], [5]. This ‘burrow-rodent-burrow’ cycle may maintain the plague bacillus in some endemic foci. Moreover, the plague bacillus can persist in environmental samples even outside animal bodies. Yersin himself recovered the plague bacillus from the soil of infected houses [6], and *Y. pestis* was shown experimentally to persist for 7 and 16 months in non-sterilized and sterilized ground, respectively [7]. Recently, a soil sample naturally impregnated with the blood of a plague infected animal still contained live *Y. pestis* ≥3 weeks after the animal’s death [8]. Water bottles seeded with *Y. pestis* and stored at 26°C allowed the recovery of viable bacteria for ≥74 days [9]. Finally, there is also the threat of a bioterrorist act, leading to the deliberate spread of *Y. pestis* in the environment [10].

There is therefore a need for an easy and efficient detection of *Y. pestis* in fleas or other potentially infected environmental sources. PCR [11] and other techniques [12], [13] have been developed, but they could not be easily performed under field conditions in endemic plague foci. A simple and rapid immunoassay test (F1 dipstick) has been developed and validated in Madagascar [14], and proved very useful for plague diagnosis. The F1 dipstick, which detects the F1 antigen, has nonetheless several limitations: (i) possible cross reactions with other antigens [15], (ii) no detection of natural [16] or genetically engineered [17] F1-negative virulent *Y. pestis*, and (iii) not usable for *Y. pestis* detection from fleas or other environmental sources since the F1 antigen is produced mainly at the body temperature of 37°C [18].

The plasminogen activator protein (PLA) encoded by the *Y. pestis*-specific pPla plasmid [18] may represent a promising mean to circumvent these problems, since previous studies showed that PLA-specific monoclonal antibodies (mAb) do not react with other Gram-negative bacteria, but detected *Y. pestis* strains grown at both 28°C and 37°C [19].

The aim of this study was to have in hands immunoassays targeting the PLA antigen as alternative rapid tests for plague diagnosis in humans or rodents, and for the fast and easy detection of *Y. pestis* from environmental sources and non-mammalian animal species such as fleas.

## Materials and Methods

### Strains and Growth Conditions

The bacterial strains used in this study are listed in Table S1. All strains were grown in Luria Bertani Broth (LB) at 28°C (*Yersinia*) 25°C (*E. pyrifoliae*), 30°C (*S. enterica*), or 37°C (*E. coli*), unless otherwise stated.

### Ethics Statement

All experiments were performed in compliance with the French and European regulations on care and protection of Laboratory Animals (EC Directive 86/609, French Law 2001-486, June 6, 2001) with agreement n°91–416 delivered to S. Simon by the French Veterinary Services and CEA agreement D-91-272-106 from the Veterinary Inspection Department of Essonne (France). Mice were sacrificed by CO2 inhalation.

### Recombinant His-tagged PLA Production, Purification and Refolding

The *pla* gene of *Y. pestis* was synthesized (Genecust) based on the published sequence of strain CO92, and cloned into the *Nde*I and *Xho*I restriction sites of the IPTG inducible pET22b vector (Novagen), allowing insertion of a poly-histidine tag sequence at the 3′ end of the gene. The pET22b-*pla* recombinant plasmid (Table S1) was used to transform competent *E. coli* BL21 cells, hereafter referred to as BL21(pla). One transformant was grown in 500 ml of LB with 100 µg/ml ampicillin at 37°C until the OD_600 nm_ reached 0.4. IPTG (1 mM) was then added to the culture that was incubated overnight at 37°C with shaking. The culture was pelleted by centrifugation at 2,000×g for 20 min at 4°C. After suspension of 5 g of the pellet in 15 ml of Tris buffer (Tris-HCl 0.1 M pH8) containing 1 mM of proteases inhibitor (AEBSF, Interchim), the bacterial suspension was sonicated (2 pulses of 30 sec), and centrifuged at 6,000×g for 30 min at 4°C. The pellet containing the inclusion bodies was suspended in 15 ml of solubilizing buffer (sodium phosphate 20 mM pH 7.4, urea 8 M, NaCl 0.5 M, Imidazole 10 mM) and was allowed to dissolve for 1 h at 4°C. After centrifugation at 6,000×g for 30 min at 4°C, the supernatant was recovered. The pellet was dissolved in 8 ml of solubilizing buffer and sonicated for 1 min before centrifugation at 6,000×g for 30 min at 4°C. The supernatants of the 2 previous centrifugations were pooled and AEBSF (1 mM final concentration) was added before loading on a 3 ml Ni-NTA agarose affinity resin (Chelating sepharose FastFlow, GE Healthcare). After a 1 h incubation at room temperature (RT) and washing with 10 ml of solubilizing buffer, elution of the His-tagged protein was performed with 4 ml of solubilizing buffer. A second cycle of binding/elution from Ni-NTA resin was performed using the unretained fraction of the first binding. The eluted fractions were pooled and dialyzed twice in 2 L of renaturation buffer (20 mM Tris-HCl, 1 M NaCl, 0.5% W/V detergent SB3-12 (N-Dodecyl-N,N-dimethyl-3-ammonio-1-propanesulfonate (Sigma)) [20], [21]. Purified recombinant PLA was allowed to refold in this renaturation buffer (supplemented with 2 mM AEBSF for 24 h at 40°C. Protein concentration was measured by absorbance at 280 nm and the purity was assessed by SDS PAGE (Phast system, GE Healthcare).

### SDS PAGE and Western Blotting

Total proteins or purified PLA were suspended in Laemmli buffer containing 0.1% SDS and kept at RT or denatured for 5 min at 95°C. After SDS-PAGE for 1 h at 200 V in a 13% gel, the proteins were stained with Coomassie blue. Molecular weight markers were Precision Plus Protein Standards (Bio-Rad).

For Western blotting, bacterial suspensions were suspended in Laemmli buffer containing 2% SDS, denatured for 5 min at 95°C, and subjected to SDS-PAGE for 2 h at 120 V in a 12% gel. Proteins were transferred overnight at 25 V onto a PVDF membrane (Amersham Biosciences). Saturation, washes and incubations with antibodies were performed using the SNAP i.d Protein Detection System (Millipore). Briefly, the membrane was blocked with skimmed dry milk (0.25%) in PBS-tween 0.1% buffer (PBST). The Pla35 mAb was diluted to 20 µg/ml in blocking buffer and allowed to react for 10 min at RT with the proteins transferred to the membrane. The membrane was subjected to three washes in PBST, and incubated for 10 min at RT with Horse Radish Peroxidase (HRP) labeled polyclonal goat anti-mouse immunoglobulins (ThermoFisher). After three washes in PBST, protein bands were detected by chemiluminescence (ECL, Amersham Biosciences), using a Versadoc imaging system (Bio-Rad).

### Production of Monoclonal Antibodies against PLA

Ten weeks-old female BALB/c mice were immunized monthly for 4 months by injection into the foot pad of 50 µg of refolded PLA with Alum adjuvant. Mice were bled before the first immunization (S0, used as negative control) and two weeks after the second and third immunizations (S2 and S3, respectively). The polyclonal anti-PLA response was evaluated by ELISA, using BL21(*pla*) as coated antigen. Two mice presenting the highest anti-PLA antibody titers were selected for preparation of monoclonal antibodiesand given a daily intravenous booster injection of 30 µg of refolded PLA for three days. Two days after the last boost, hybridomas were produced by fusing spleen cells with NS1 myeloma cells, as previously described [22]. The hybridomas’ culture supernatants were screened for the presence of anti-PLA antibodies by ELISA. Selected hybridomas were subsequently cloned by limiting dilution. mAbs were obtained after inducing ascites in BALB/c mice, and further purified using caprylic acid precipitation [23].

### Pepscan Analysis

A collection of 85 peptides of 9 amino acids each, overlapping each other by 7 residues and covering the five extracellular loops of PLA and adjacent sequences was synthesized using the SPOT-synthesis method [24] on an AutoSpot apparatus (Intavis AG). They were then linked to a derivatized cellulose membrane (Intavis AG) and probed for 30 min with 5 µg/ml Pla45 and Pla35 mAbs. After 3 washes in PBST, membranes reacted for 30 min at RT with HRP labeled goat anti-mouse immunoglobulins (ThermoFisher). Protein spots were detected by chemiluminescence (ECL, Amersham Biosciences).

### Enzyme Immunoassays

To label antibodies with biotin, mAbs in 0.1 M borate buffer pH 9 were incubated at a 1∶20 molar ratio with biotin-N-hydroxysuccinimide ester (Sigma) dissolved in anhydrous DMF. The reaction was stopped after 30 min at RT by addition of 1 M Tris-HCl pH 8 for 1 h at RT. The conjugate diluted in EIA buffer (0.1 M phosphate buffer pH 7.4 containing 0.15 M NaCl, 0.1% bovine serum albumin and 0.01% sodium azide) was then stored at −20°C until use. Labeling of antibodies and streptavidin using acetylcholinesterase (AChE) or biotin was performed as described in [25]. To label streptavidin, Thiol groups were first introduced by reaction of its primary amino group with N-succinimidyl-S-acetylthioacetate (SATA) in alkaline medium. Streptavidin-SATA was subsequently coupled to AChE-SMCC [25].

To titrate anti-Pla IgG in mouse sera, *E. coli* BL21(*pla*) and BL21 (used as negative control) were grown overnight in LB at 37°C without IPTG. After centrifugation for 10 min at 2,000×g at 4°C, pellets were suspended in water to a concentration of 10^8^ cfu/ml. 100 µl of this suspension was distributed in each well of 96-well microtiter plates (Maxisorp, Nunc) and allowed to dry overnight at RT. After saturation with 200 µl of EIA buffer and 3 washing cycles with the washing buffer (0.01 M potassium phosphate pH 7.4), 100 µl of 10 fold serial dilutions (from 10^−2^ to 10^−5^) in EIA buffer of mouse sera or of each culture supernatant from 96-well culture plates were transferred into the microtiter plates coated with the bacteria. The plates were incubated for 18 h at 4°C and washed before the addition of 100 µl of AChE-labeled anti-mouse Ig(G+M) (Jackson ImmunoResearch) conjugate (2 Ellman units [EU]/ml) to each well. After 3 h incubation at RT followed by three washing cycles, 200 µl of Ellman’s reagent [26] were added, and the absorbance was measured at 414 nm after 30 min.

To evaluate the best mAbs pairs to be used in a two-site immunometric test, a combinatorial analysis was carried out using each mAb either as capture or conjugate Ab, using BL21(*pla*) as target. Immobilization of the capture mAb in microtiter plates was performed by distributing 120 µl/well of the antibody at a concentration of 10 µg/ml in potassium phosphate buffer 0.05 M pH 7.4 and incubating the plates overnight at RT. The plates were then emptied, saturated with EIA buffer for 18 h at 4°C, and kept until use. Plates were then washed 3 times with washing buffer (0.01 M potassium phosphate pH 7.4 containing 0.05% Tween 20). Overnight cultures of BL21(*pla*) were centrifuged for 10 min at 2,000×g and the pellets were suspended in EIA buffer. 100 µl of a suspension containing 10^9^ cfu/ml were distributed in duplicates in the wells of microtiter plates precoated with the various capture antibodies to be tested. EIA buffer was used as a negative control. After an overnight incubation at 4°C and three washing cycles, 100 µl of biotin-labeled tracer mAb (500 ng/ml) were added and the microtiter plates were incubated at RT for 4 h. Plates were washed, and after addition of 100 µl/well of AChE-labeled streptavidin conjugate (2 EU/ml), they were kept at RT for 1 h. Plates were then washed, 200 µl of Ellman’s reagent was added, and absorbance at 414 nm was read after 30 min incubation at RT. Specific signals were determined on duplicate bacterial suspensions, while non-specific adsorption was determined with duplicate wells containing the EIA buffer. Other EIA tests were done according to the procedure described above with Pla45 as the capture mAb, and used various bacterial suspensions at different dilutions as antigens, and either a biotinylated Pla35 (Pla 35*) or a biotinylated anti-F1 (anti-F1*) mAb kindly provided by F. Nato (Institut Pasteur) as tracer antibody.

The optimized enzyme immunoassay consisted in distributing 50 µl of the antigenic sample together with 50 µl of AChE-labeled mAb (10 EU/ml final concentration) in duplicates into the micro wells of an ELISA plate precoated with Pla45 (capture antibody). The plates were centrifuged for 5 min at 1,000×g and incubated for 3 h at 30°C. They were washed 3 times with washing buffer (0.05 M potassium phosphate pH 7.4) before adding 200 µl/well of Ellman’s reagent. The plates were kept at RT for 30 min before absorbance measurement at 414 nm. The limit of detection (LoD) was calculated as the amount of bacteria giving an absorbance equivalent to the mean of negative controls plus 4 standard deviations of these negative controls, allowing 99.9% confidence.

### Lateral Flow Immunoassays

The colloidal-gold-labeled Pla35 mAb probe and the strips (0.5 cm width and 4.5 cm length) were prepared as previously described [27]. 100 µl of bacterial suspensions in analysis buffer (0.1 M potassium phosphate buffer, pH 7.4, containing 0.1% BSA, 0.15 M NaCl, and 0.5% Tween 20) were mixed with 10 µl of colloidal-gold-labeled antibodies (20 µg/ml) in the wells of a 96-well microtiter plate. After 10 min incubation of the mixture with shaking at 20°C in the dark, the strips were inserted into the wells. The capillary migration lasted for about 15 min.

The F1-dipstick was kindly provided by M. Rajerison (Institut Pasteur, Madagascar), and was used as previously described [28].

## Results

### Purification of a PLA Protein Produced by Recombinant *Escherichia coli*

To produce the large amounts of purified PLA protein necessary to immunize mice, the *pla* gene of *Y. pestis* CO92 was cloned into the IPTG inducible pET22b plasmid, generating a *pla* gene carrying a poly-histidine tag sequence at its 3′ end. The recombinant pET22b-*pla* vector was then introduced into *E. coli* BL21, yielding BL21(*pla*) (Table S1). Based on the three dimensional structures of PLA [29] and its homolog OmpT of *E. coli* [30], the poly-histidine tag, located at the C-terminal end of the recombinant protein should be in the periplasmic space of the bacteria. This was confirmed by the fact that an anti-poly-Histidine antibody did not recognize intact BL21(*pla*) bacteria in ELISA, whereas lyzed bacteria reacted with this antibody (data not shown). The functionality of the recombinant PLA was tested using the rabbit plasma coagulation test [31]. After IPTG induction, incubation of BL21(*pla*) with rabbit plasma gave rise to the formation of a clot, while the BL21 strain used as control did not (data not shown). In the absence of IPTG induction, a clot was also visible with the BL21(*pla*) strain, indicating a certain level of *pla* expression in the absence of IPTG induction. The fact that IPTG induction somehow decreased clot formation argued for the retention of large amounts of the protein inside inclusion bodies when *pla* was over expressed.

The His-tagged PLA present in the inclusion bodies was then purified by passage through an Ni-NTA sepharose resin. SDS PAGE and Coomassie blue staining of the elution product from the column showed that it contained almost exclusively the recombinant His-tagged PLA protein (Figure S1 A). Outer-membrane proteins are known to have an electrophoretic mobility that varies with their folding [32], [33]. The recombinant PLA was then treated with a zwitterionic detergent to facilitate its refolding (adapted protocol from [21]). Using mild denaturation conditions (low concentration of SDS: 0.1%), the treated form presented a higher electrophoretic mobility in SDS PAGE as compared to the unfolded forms obtained after heat denaturation (Figure S1 B), indicating that the PLA preparation was refolded.

### Production and Selection of the Most Efficient Pairs of Anti-PLA mAbs

After fusion, a total of 958 hybridomas were obtained and the corresponding culture supernatants were screened by ELISA for the presence of specific antibodies directed against PLA. Most of the antibodies were not PLA-specific, as they recognized both BL21(*pla*) and BL21 (for example clones Pla25, Pla32, Pla41 and Pla59 on Figure S2). In contrast, some antibodies reacted only with BL21(*pla*) (Pla26, Pla27, Pla35 and Pla45 on Figure S2) and were selected for their specificity. In total, 26 hybridomas presenting a strong signal with BL21(pla) and at worst for some a weak binding to control BL21 were sub-cloned by limiting dilutions. These final sub-clones were used to produce ascitic fluids from which the 26 monoclonal antibodies were purified.

**Figure 1:**
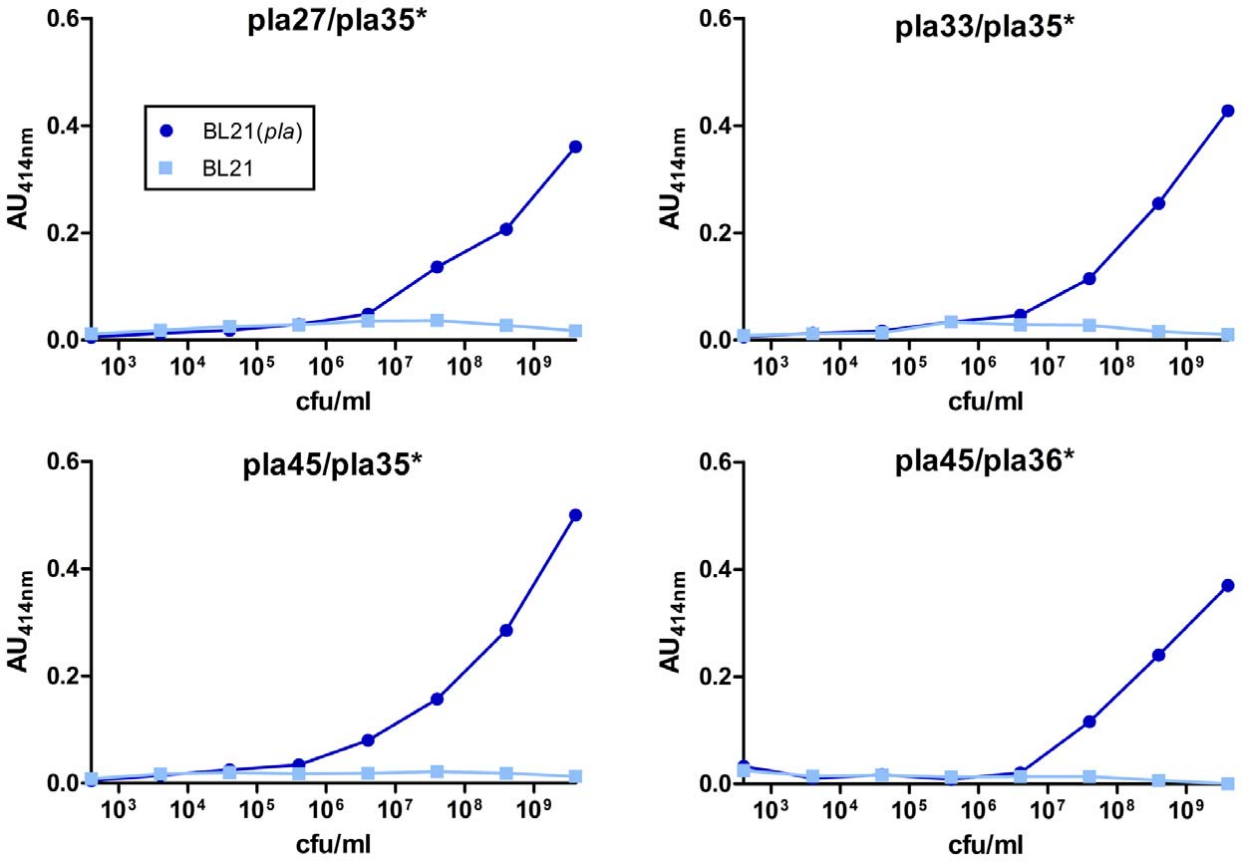
Test of the four most sensitive mAbs pairs against BL21(*pla*). Sensitivity in two-site immunoassays was determined using 10 fold serial dilutions of BL21(*pla*) or BL21 as negative control. The * indicates the tracer antibody.

To develop a sandwich ELISA, combinatorial analyses were performed, in search for the best pairs of antibodies to be used in a two-site immunometric test. Each of the 26 mAbs was tested both after immobilization on solid phase (capture antibody) and as a biotin-labeled conjugate (tracer antibody), yielding 676 combinations. An example of the results of 360 of these combinations is shown in Table S2. Thirteen pairs of mAbs gave a strong signal (Absorbance at 414 nm (AU_414_)>0.3) in sandwich immunoassays with a high concentration of BL21(*pla*) used as antigen. After testing serial dilutions of BL21(*pla*), 4 mAb pairs (Pla27/Pla35*, Pla33/Pla35*, Pla45/Pla35* and Pla45/Pla36*) displayed the highest sensitivity (Figure 1)and the best of them (Pla45/Pla35*) was selected.

### The Pla45/Pla35* mAb Pair Recognizes All Natural *Y. pestis* Strains Tested

**Figure 2:**
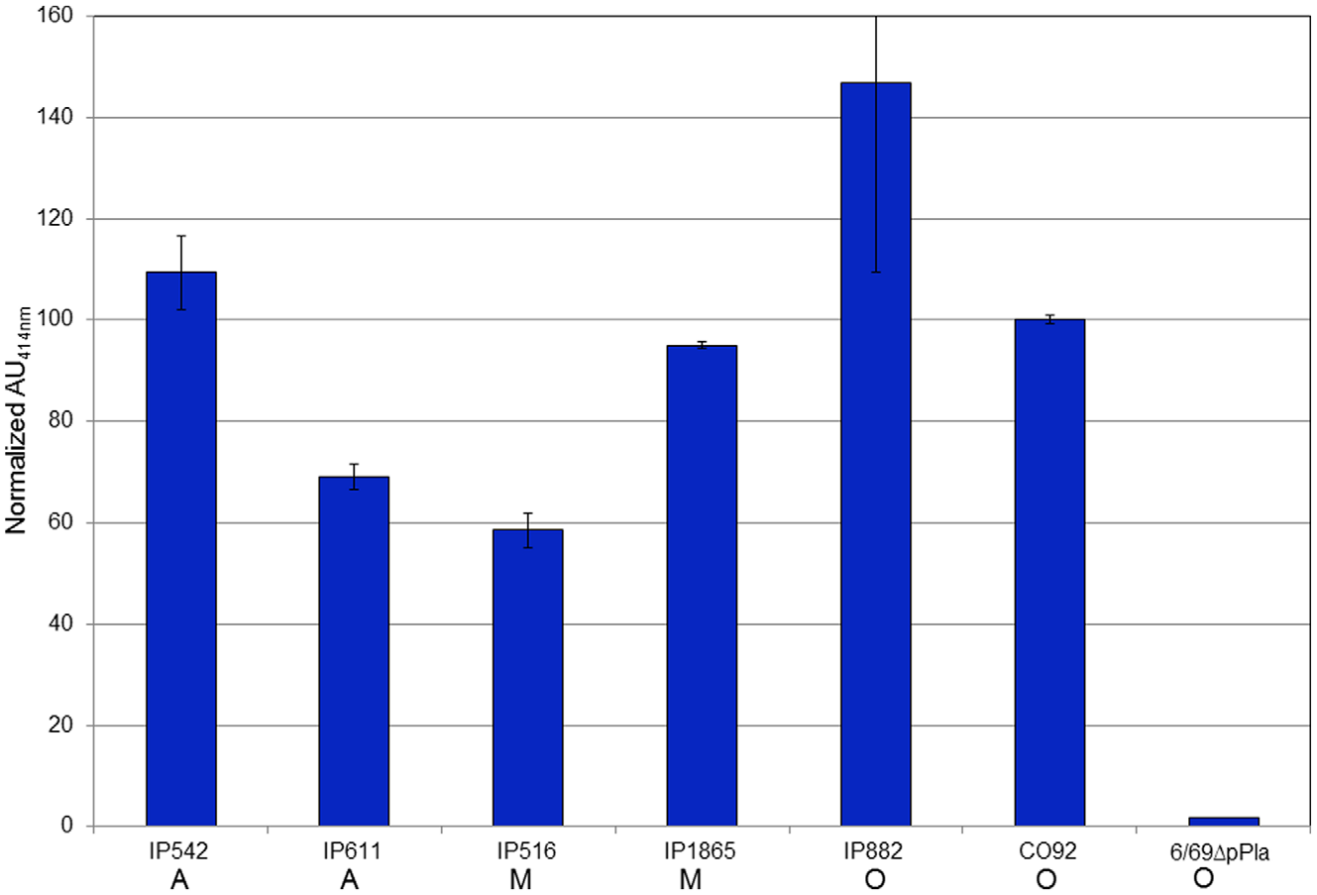
Detection of *Y. pestis* strains of various biovars by the Pla45/Pla35 * **immunoassay.** Six different strains of *Y. pestis* were grown at 28°C and 2×10^7^ cfu/ml of each strain were used as antigen in the sandwich immunoassay. Absorbances at 414 nm were normalized using the absorbance of CO92 as reference (100%). Biovars were A: Antiqua, M: Medievalis and O, Orientalis. A *Y. pestis* strain cured of pPla (6/69ΔpPla) was used as a negative control.

The capacity of the Pla45/Pla35* mAb pair to recognize a native form of PLA anchored in the bacterial outer membrane, and its comprehensiveness for the species *Y. pestis* were evaluated using a panel of six strains belonging to the most common biovars (Antiqua, Medievalis and Orientalis). Despite some strain-to-strain variations in the level of absorbance, all six *Y. pestis* strains tested were recognized in the Pla45/Pla35* sandwich immunoassay, while the signal obtained with the 6/69ΔpPla *Y. pestis* control strain remained at the background level (Figure 2). Our results thus indicate that the Pla45/Pla35* mAb pair efficiently recognizes a natural form of PLA exposed at the surface of all natural *Y. pestis* isolates tested.

### The Epitopes Recognized by Pla45 and Pla35 are Unique to PLA

**Figure 3:**
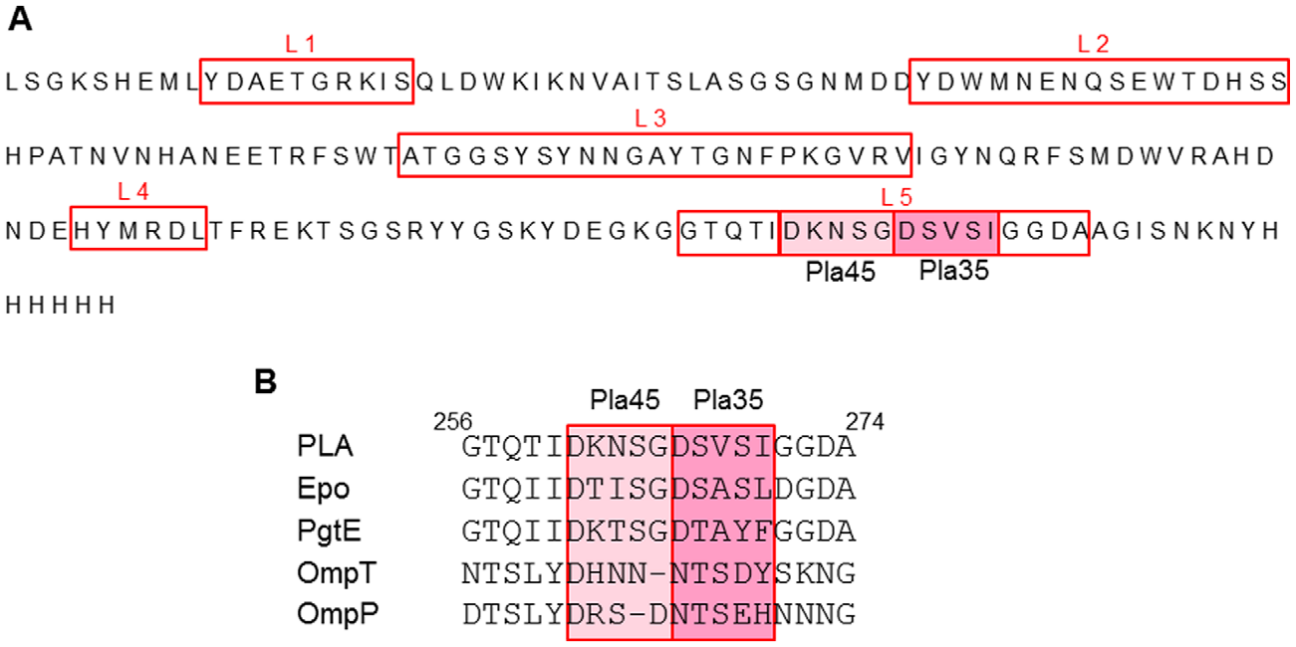
Epitope mapping of Pla45 and Pla35 mAbs. (**A**) Pepscan epitope mapping of Pla45 and Pla35 mAbs was performed with synthetic peptides covering the 5 extracellular loops (L1 to L5) and the amino acids bordering these loops in PLA. The epitopes recognized by Pla45 are shown in a pale pink box, and those recognized by Pla35 in a darker pink box. (**B**) Amino acid sequence alignment of loop 5 from PLA, Epo, PgtE, OmpT and OmpP. Epitopes recognized by Pla45 and Pla35 mAbs on PLA, and the corresponding epitopes on the other molecules are in pink boxes.

The above results suggested that the epitopes recognized by Pla45 and Pla35 are located on the outer regions of the protein in its natural anchored and folded conformation. To further characterize these epitopes, a 178 amino acid sequence covering the 5 extracellular loops of PLA (L1 to L5) [29], [30] and the amino acids bordering these loops were analyzed using a synthetic peptide array. When probed with the Pla45 and Pla35 mAbs by immunoblotting, adjacent linear epitopes of 5 residues located in loop 5 were identified, i.e. 261-DKNSG-265 for Pla45, and 266-DSVSI-270 for Pla35 (Figure 3A).

Several enterobacteria present in the environment are known to produce a protein sharing some sequence identities and structural homologies with PLA, i.e. PgtE of *Salmonella enterica* serovar Typhimurium, Epo of *Erwinia pyrifoliae,* and OmpT and OmpP of *E. coli* [30], [34]. Sequence alignment of PLA with its homologs in this region evidenced differences in their amino acid sequences (Figure 3B), suggesting that these regions should not be recognized by the Pla45 and Pla35 mAbs.

### The Pla45/Pla35* mAb Pair is PLA-specific

**Figure 4:**
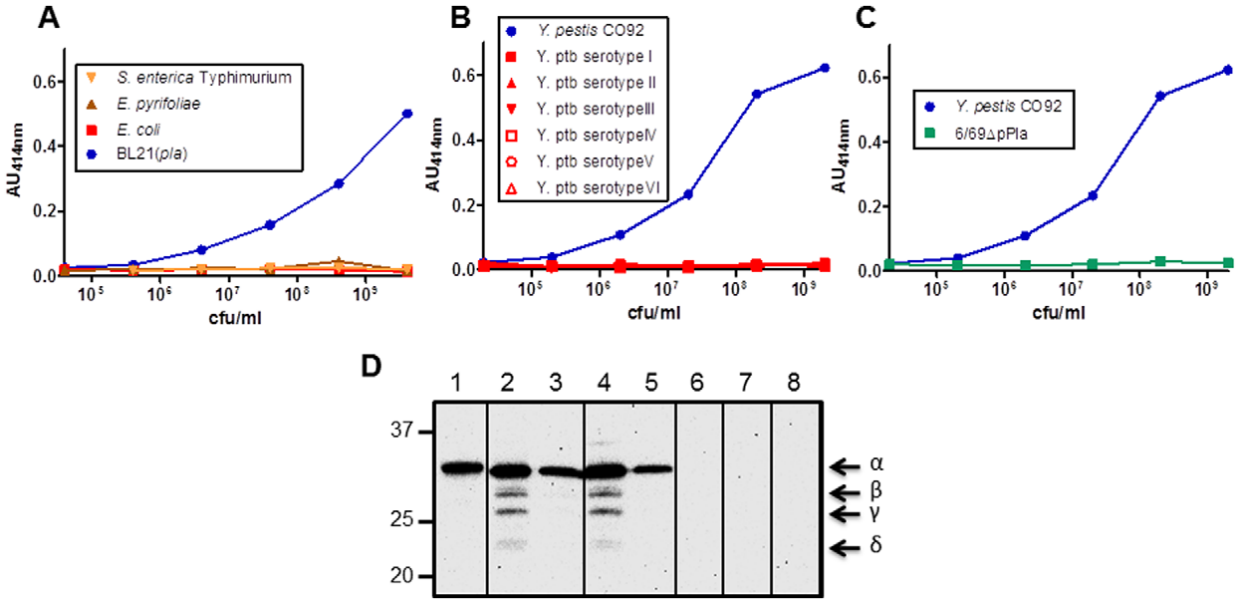
Specificity of the Pla45/Pla35* mAb pair for PLA. (**A**) Sandwich immunoassay against *Salmonella enterica* serovar Typhimurium, *Erwinia pyrifoliae*, *Escherichia coli* and BL21(*pla*). (**B**) Sandwich immunoassay against *Y. pestis* CO92 and six strains of *Y. pseudotuberculosis* of various serotypes (for serotypes I to VI). (**C**) Reactivity of the Pla45/Pla35* pair against *Y. pestis* strain CO92 harboring pPla, and strain 6/69ΔpPla cured of the plasmid. (**D**) Western-blotting with Pla35 against recombinant PLA (lane 1, 1 µg), whole cell extracts of *Y. pestis* CO92 at a concentration of 4×10^6^ cfu/well (lane 2), or 4×10^5^ cfu/well (lane 3), IP516 at a concentration of 4×10^6^ cfu/well (lane 4), or 4×10^5^ cfu/well (lane 5), and 6/69ΔpPla (lane 6, 4×10^7^ cfu/well), *Y. pseudotuberculosi*s IP32953 (lane 7, 4×10^7^ cfu/well), and *E coli* BL21 (lane 8, 4×10^7^ cfu/well). Numbers on the left indicate the molecular weight markers (in kDa). Greek letters on the right indicate the various forms of PLA.

To further determine the specificity of the Pla45/Pla35* pair for PLA of *Y. pestis*, three enterobacteria producing a PLA homolog (*S. enterica* serovar Typhimurium, *E. pyrifoliae,* and *E. coli*) were subjected to the Pla45/Pla35* sandwich immunoassay. As shown in Figure 4A, BL21(*pla*), but none of the other enterobacteria tested, reacted with the Pla45/Pla35* mAbs.

*Y. pestis* is genetically and phenotypically closely related to *Y. pseudotuberculosis* and therefore most of their antigens are common to the two species [35], [36]. The Pla protein is encoded by a gene present on a 9.5 kb plasmid (pPla) acquired by *Yersinia pestis* after its divergence from *Yersinia pseudotuberculosis*. We thus also wanted to determine whether Pla45/Pla35* would recognize some epitopes shared by these two organisms. For this purpose, *Y. pestis* CO92 [37] and six strains of *Y. pseudotuberculosis* of various serotypes were used as antigens in the sandwich ELISA test. The Pla45/Pla35* pair did recognize *Y. pestis* CO92, but did not react with the various *Y. pseudotuberculosis* strains tested (Figure 4B). Finally, a *Y. pestis* strain cured of the pPla plasmid (6/69ΔPla) was not detected with the sandwich immunoassay (Figure 4C), confirming the specific targeting of PLA.The specificity of the Pla35 tracer antibody was also evaluated in western-blot experiments. This antibody reacted with the purified recombinant PLA from *E. coli*, which migrated as a product of slightly higher molecular weight than the natural PLA, due to the presence of the poly-histidine tag (Figure 4D, lane 1). Pla35 also recognized specifically the native PLA protein produced by two different strains of *Y. pestis* (Figure 4D, lanes 2–5). In the two wells containing the lowest bacterial numbers, only the major form of the protein (α-form) was visible (lanes 3 and 5), while at higher bacterial numbers, the four molecular forms of PLA (α, β, γ and δ-PLA) [38] were detected (lanes 2 and 4). No signal was visible with cell extracts of *Y. pestis* cured of pPla (lane 6), or with *Y. pseudotuberculosis*, *Y. enterocolitica* or *E. coli* (Figure 4D, lanes 7–9). Altogether these results indicate that the Pla45/Pla35* pair of mAbs is specific for PLA.

### Detection of *Y. pestis* by the Pla45/Pla35* Sandwich Immunoassay is not Temperature-dependent

**Figure 5:**
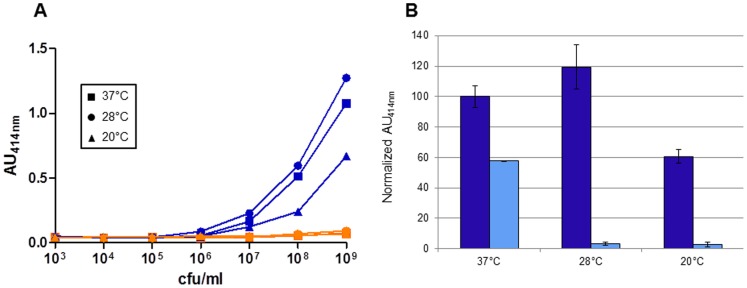
Impact of the growth temperature on PLA detection by sandwich ELISA. (**A**) *Y. pestis* CO92 (blue lines) and *Y. pseudotuberculosis* IP32953 (orange lines) were grown at three temperatures: 37°C (▪), 28°C (•) and 20°C (▴). Ten-fold serial dilutions of the cultures were used as antigens for the Pla45/Pla35* sandwich ELISA. (**B**) Comparison of *Y. pestis* detection using an anti-PLA or an anti-F1 tracer antibody. 10^9^ cfu/ml of *Y. pestis* cultivated at three different growth temperatures (20°C, 28°C and 37°C), were incubated with Pla45 as capture antibody, and with either Pla35* (dark blue), or anti-F1* (light blue) conjugates.

One of the major aims for the development of a new *Y. pestis* test was to gain the capacity to detect the bacteria at temperatures usually found in the environment. For this purpose, *Y. pestis* CO92 was cultured at three temperatures (20°C, 28°C and 37°C), along with *Y. pseudotuberculosis* IP32953 [39] used as control. As shown in Figure 5A, *Y* *. pestis* was recognized by the Pla45/Pla35* pair at the three temperatures, while the *Y. pseudotuberculosis* signal remained at the background level, whatever the growth temperature. *Y. pestis* cultured at 28°C gave a signal as strong (or even slightly stronger) as at 37°C, and a signal was still detected at 20°C, although of lower intensity (Figure 5A).

To compare the effect of growth temperature on the detection of *Y. pestis* with anti-PLA or conventional anti-F1 antibodies, an immunoassay was designed using the Pla45 mAb as capture antibody and either Pla35* or an anti-F1* biotinylated mAb as a conjugate antibody in the sandwich format. As found above, *Y. pestis* CO92 was recognized by the anti-PLA tracer antibody at the three temperatures (Figure 5B). Not unexpectedly, the anti-F1 tracer antibody reacted with bacteria grown at 37°C, but the signal obtained on bacteria cultivated at 28°C and 20°C could be hardly distinguished from the background level. These results demonstrate that PLA is a much better target than F1 for the detection of *Y. pestis* grown outside a mammalian host.

### Optimization of the Conditions and Sensitivity of the Sandwich PLA-ELISA

Our results indicated that the sandwich PLA-ELISA using the Pla45/Pla35* mAb pair allows a specific and reliable recognition of *Y. pestis* strains grown at various temperatures, and therefore that it could be a promising test for the environmental detection of this organism. To optimize it, we tested different conditions aiming at simplifying the procedure and decreasing its duration, while retaining its performances. This resulted in a modification of the initial test as follows: (i) the biotinylated tracer antibody was replaced by an antibody directly labeled with AChE, thus reducing non-specific binding and removing the AChE streptavidin-labeling step, (ii) the bacteria and the AChE-labeled Pla35 antibody were diluted in LB Broth instead of EIA buffer to prevent bacterial lysis, (iii) the bacteria and the tracer mAb were mixed and incubated at the same time instead of being incubated sequentially, thus removing another step, (iv) incubation of the bacteria and the tracer mAb with the solid phase were performed at 30°C instead of 4°C to accelerate the binding, and (v) a centrifugation step was introduced to favor the binding of bacteria to the capture Pla45 antibody and to shorten the incubation time (3 h instead of 16 h). These new conditions resulted in a one-step assay of 4 h instead of a 3-step test of 20 h.

**Figure 6:**
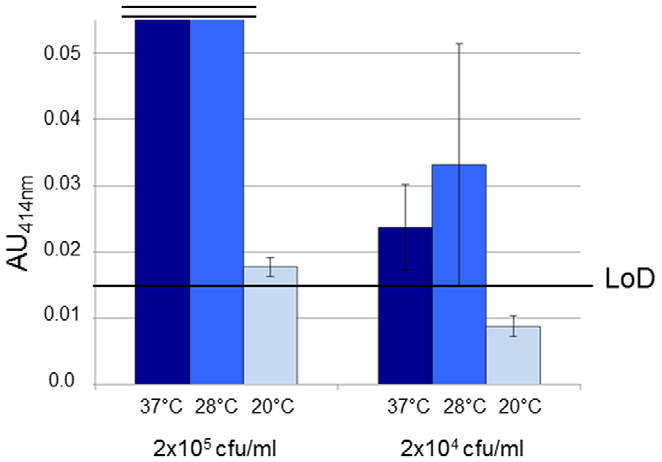
Sensitivity of the optimized Pla45/Pla35* immunoassay. *Y. pestis* CO92 was grown at 37°C (dark blue), 28°C (medium blue), or 20°C (light blue), and subjected to the optimized sandwich ELISA. The graphs represent the lowest AU values. The limit of detection (LoD) is represented by a horizontal black line. Vertical lines represent the standard deviation of duplicates.

Using the initial sandwich ELISA procedure, the sensitivity of the test was ≥10^6^ cfu/ml for BL21(*pla*) (Figure 1) and for *Y. pestis* CO92 grown at 28°C (Figure 4A). This sensitivity was significantly increased with the optimized sandwich PLA-ELISA. Indeed, the limit of detection of *Y. pestis* CO92 dropped to 2×10^4^ cfu/ml (i.e. 10^3^ cfu/well) when the strain was grown at 28°C or 37°C, and to 2×10^5^ cfu/ml (10^4^ cfu/well) for a strain grown at 20°C (Figure 6).

### Development of a Lateral Flow Immunoassay for *Y. pestis* Detection under Field Conditions

Although simplified and optimized, the sandwich PLA-ELISA might not be easy to use under field conditions in plague endemic foci. In order to get a rapid and easy handling test, we decided to develop a lateral flow immunoassay (dipstick). The two best characterized antibodies, Pla35 and Pla45, were used both as capture and tracer Abs and the capacity of the combinations Pla45/Pla35*, Pla45/Pla45*, Pla35/Pla35* and Pla35/Pla45* to detect *Y. pestis* by lateral flow immunoassay was compared. The best results were obtained with the Pla35/Pla35* combination (data not shown). The performances of this dipstick in terms of specificity, sensitivity, comprehensiveness and capacity to detect bacteria grown at different temperatures were further evaluated.

**Figure 7:**
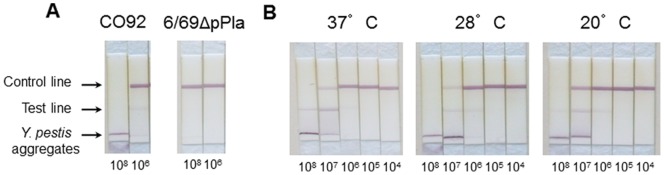
Lateral flow immunoassays. (**A**) Specificity of the PLA-dipstick for *Y. pestis* harboring pPla. Both CO92 and 6/69ΔpPla were grown at 28°C and serially diluted. The bacterial suspensions were incubated for 10 min with colloidal gold-labeled Pla35 mAb, and the PLA-dipsticks were then dipped for 30 min into 100 µl of the bacterial suspensions for upward migration of the liquid. Numbers below the dipsticks indicate the number of cfu/well. (**B**). Influence of the temperature of culture on CO92 detection. Bacteria were grown at 20°C, 28°C or 37°C, and the tests were performed as described in (A).

Whatever the antibody combinations, at high bacterial concentrations (10^8^ cfu/well corresponding to 10^9^ cfu/ml), a specific band was detected in the lower part of the membrane (Figure 7A). This band resulted from the formation of aggregates composed of tracer antibodies bound to intact bacteria that were too large to migrate along the membrane. At lower bacterial concentrations, the intensity of this high molecular weight band decreased while the intensity of the bands corresponding to the test line and control line increased (Figure 7A).

The PLA-dipstick was found to be as specific as the PLA-EIA, as none of the Gram-negative bacteria producing a PLA homolog (*E. coli*, *S. enterica* and *E. pyrifoliae*) were detected, even at high bacterial concentrations (Table 1). Similarly, no reaction was observed with the two *Y. pseudotuberculosis* strains tested (Table 1) or with the *Y. pestis* strain cured of pPla (Figure 7A and Table 1).

**Table 1:** Specificity, sensitivity and comprehensiveness of the PLA-dipstick.

	Species	Strain	Biovar	cfu/well				
				10^8^	10^7^	10^6^	10^5^	10^4^
**Specificity**								
	*E. coli*	BL21	NA	−	−	−	−	−
	*S. enterica*	CIP104474	NA	−	−	−	−	−
	*E. pyrifoliae*	CIP106111	NA	−	−	−	−	−
	*Y. pseudotuberculosis*	IP31629	NA	−	−	−	−	−
	*Y. pseudotuberculosis*	IP33434	NA	−	−	−	−	−
	*Y. pestis*	6/69ΔpPla	Orientalis	−	−	−	−	−
**Comprehensiveness/sensitivity**								
	*Y. pestis*	CO92	Orientalis	**+**	**+**	**+**	−	−
		IP882	Orientalis	**+**	**+**	**+**	−	−
		IP542	Antiqua	**+**	**+**	**+**	−	−
		IP611	Antiqua	**+**	**+**	**+**	−	−
		IP516	Medievalis	**+**	**+**	**+**	−	−
		IP1865	Medievalis	**+**	**+**	**+**	−	−

Each strain was cultured at its optimal growth temperature (see Materials and methods). The bacterial suspensions were adjusted to ca. 10^9^ cfu/ml and serially diluted. 100 µl of these suspensions were used for the dipstick assays.

NA: not applicable.

**+**: positive test line and/or *Y. pestis* aggregates line.

**−**: negative test line and *Y. pestis* aggregates line.

All six *Y. pestis* strains analyzed gave a signal with the PLA-dipstick (Table 1), indicating that this test is as comprehensive as the PLA-EIA. However, the sensitivity of the PLA-dipstick was lower than that of the PLA-EIA, as its detection limit was approximately 10^6^ cfu/well (Table 1). This detection limit was similar for all *Y. pestis* strains tested (Table 1).

The PLA-dipstick detected *Y. pestis* CO92 grown at 37°C, 28°C and 20°C (Figure 7B). At these three temperatures, the detection limit was 10^6^ cfu/well, although the intensity of the band at this concentration was slightly fainter for bacteria grown at 37°C (Figure 7B). The F1-disptick [14] was 10-fold more sensitive (detection limit of 10^5^ cfu/well) than the PLA-dipstick for *Y. pestis* CO92 grown at 37°C, but it did not detect bacteria grown at 28°C, even at the highest concentration of 10^8^ cfu/well (data not shown). Finally, the PLA-dipstick, but not the F1-dipstick, detected as efficiently the *Y. pestis* CO92 mutant unable to synthesize the F1 antigen as the wild type CO92 strain (data not shown).

## Discussion

The identification of the etiological agent of plague [6], its reservoir and its vector [40] at the end of the 19th century, and the subsequent advent of effective therapies allowed the implementation of preventive and control measures, as well as the treatment of plague patients. Although these major discoveries have led to a dramatic drop in the number of human cases reported worldwide, plague has not been eradicated. On the opposite, the disease has re-emerged since the 1990’s in countries where no human cases were reported for decades. This has been the case for instance in Zambia in 1993, in a region where no plague cases were observed over the past 39 years [41], in India, where a major outbreak of pneumonic plague occurred in 1994 after 30 years of silence [42], in Algeria in 2003 [43] after five decades during which the disease was considered as extinct, and even more recently in Libya in 2009 after 25 years without any human cases of plague reported [44]. Therefore plague is now considered as a re-emerging disease [1]. This apparent reemergence is in great part explained by the fact that the presence of the disease is noticed only when human cases are observed [45], [46]. However, humans are only accidental hosts, and the plague agent may persist for long period of times within its natural ecological niche without causing any human infections. Vast territories of plague foci exist for instance in central Asia (Kazakhstan, China), in which human cases are rare because of limited contacts between rodent reservoirs and humans [47]–[50] and because of relatively high host-specificity of some fleas species carrying the disease. It is thus of key importance to perform epidemiological surveillance of the disease, at least in plague endemic foci, to monitor plague activity and to implement control measures as soon as the level of the risk increases. This is far from being systematically done in endemic areas because the workload is heavy, as it requires capturing and dissecting rodents, collecting their fleas, and performing serological and bacteriological analyses on these samples. Furthermore, the search for a *Y. pestis* reservoir in the environment (and in particular in rodents’ burrows) is seldom performed today.

This workload may be significantly alleviated by a preliminary on site screening of rodent blood or organs with the F1-dipstick [14]. This test does not require any sophisticated equipment and detects positive samples within 10 minutes, thus permitting to restrict bacteriological analyses to these biological materials. However, the F1 dipstick is not applicable to the detection of *Y. pestis* in fleas or the environment because the F1 pseudo-capsule is almost essentially produced at the host temperature of 37°C [18]. There was therefore a need for a test that could detect an antigen produced not only at 37°C, but also at temperatures comprised between 21°C and 30°C, which are often found in the environment and are optimal for flea survival. We chose the PLA antigen for the development of such a test because it combines the advantages of being *Y. pestis*-specific, surface exposed, produced in significant amounts, important for virulence, and synthesized not only at 37°C but also at lower temperatures [19], [51].

In order to develop PLA-based immunometric tests, we first produced a large set of anti-PLA mAbs and screened them for their specificity and sensitivity. This allowed us to identify a mAb pair that recognized PLA epitopes predicted to be exposed at the bacterial surface, and whose amino acid sequences were different from those of PLA homologs produced by other enterobacteria commonly found in the environment. This pair of mAbs, used in a simplified PLA-EIA assay, led to the development of a one-step, 4 h-long test. However, although simplified, the PLA-EIA test still requires devices (such as a centrifuge), hardly compatible with a field application. We thus also used one of the two mAbs selected to develop a lateral flow immunoassay (PLA-dipstick) that could be handled under field conditions by untrained staff, and that gives a result in less than 30 min. At high concentrations of bacteria (≥10^7^ cfu/well), a line corresponding to bacteria/tracer antibody aggregates stuck in the lower part of the dipstick was observed. This line corresponds to a specific labeling since no such band was detected with bacteria other than *Y. pestis,* or with a *Y. pestis* strain cured of pPla. The test line (upper position on the dipstick) was also visible, but fainter because most of the mAbs were trapped in the aggregates. At lower bacterial concentrations, the lower specific test line tended to disappear in favor of the upper test line. Therefore, these two lines should be considered as specific test lines.

PLA is homologous to other omptins of Gram-negative bacteria, sharing up to 74% sequence identity with PgtE of *Salmonella enterica* serovar Typhimurium and Epo of *Erwinia pyrifoliae* [34]. Despite these common sequences, the two PLA-immunometric tests detected neither these enterobacteria, nor the genetically close *Y. pseudotuberculosis* species, demonstrating their specificity for the PLA antigen of *Y. pestis*. These two PLA-based tests recognized all six *Y. pestis* strains belonging to the three common biovars (Orientalis, Medievalis and Antiqua). The Pestoides/Microtus biovar could not be tested, as no isolates of this biovar are available in our laboratory. However, these strains are extremely rare, they are limited to a specific geographical area in central Asia, and most of them harbor a pPla plasmid [52]. It is thus expected that the PLA-EIA and PLA-dipstick assays will detect almost all, if not all *Y. pestis* isolates. Furthermore, as PLA is a key virulence determinant in most *Y. pestis* isolates [18], [34], [53]–[55], strains naturally cured of pPla or genetically engineered to delete *pla* would lose their pathogenicity and would thus be of no concern.

As the PLA-immunometric tests recognize *Y. pestis* grown at 37°C, they could be used for the diagnosis of plague in rodents and humans. The F1-dipstick is a highly valuable test for plague diagnosis under field conditions [56], and it is now widely used in countries with endemic plague foci. The PLA-dipstick had a sensitivity of ≈10^6^ cfu/well for bacteria grown at 37°C, which was 10-fold lower than that of the F1-dipstick, indicating that the latter remains the most appropriate rapid test to be performed under field conditions on rodent or human samples. However, the standard case definition of WHO states that in areas not known to be plague foci, a positive F1-dipstick alone is not sufficient for confirmation of a human plague case [57]. Indeed, although infrequent, cross-reactions between F1 and non-*pestis* antigens may exist [15], and it is essential to obtain a second positive test, independent of F1, to confirm the plague etiology in a region initially considered as plague-free. This is illustrated by the example of the pneumonic plague outbreak that occurred in a diamond mine in Zobia (Democratic Republic of Congo) in 2005 [58], [59]. Several patients presented with clinical signs of pneumonic plague and their sputum was positive with the F1-dipstick. However, the absence of *Y. pestis* isolation from the sputum samples and the occurrence of this episode in a place very distant from the known plague foci in this country casted some doubts about the presumptive plague etiology. A serological conversion observed in some patients (but again based on the detection of anti-F1 antibodies) confirmed, but only retrospectively, the diagnosis. The availability of a PLA-dipstick that can be used at the patient’s bedside in remote areas, and the possible rapid confirmation in a laboratory setting with the highly sensitive (10^3^ cfu/well for *Y. pestis* grown at 37°C) PLA-EIA assay would thus be highly valuable in this context. Furthermore, it has been shown that *Y. pestis* strains devoid of or with low levels of F1 antigen naturally occur and may have a virulence comparable to that of F1-positive strains [16], [60], [61]. These strains proved to be undetectable using a classical F1-dipstick [55]. In this case, or in the case of a genetically engineered F1-negative *Y. pestis* [17], [62], [63], a diagnostic test that does not target F1, such as the PLA-dipstick or the PLA-EIA would be of major interest.

There are accumulating data suggesting a long-term persistence of *Y. pestis* in natural reservoirs other than mammals, such as rodent burrows [4], [5], [64], soil [6]–[8], and possibly water [9]. In addition, there is a risk of an intentional spread of *Y. pestis* in the environment with the aim of using this bacterium as a biological weapon [10]. Finally, another important non-mammalian reservoir is the flea, which is a key element of the plague epidemiological cycle and persistance in the fields [65]. However, the plague agent is maintained and/or multiplies at temperatures below 37°C in these samples, and therefore it is not detectable with the F1-dipstick. There is thus a need for rapid and sensitive tests for environmental detection of *Y. pestis*. The two PLA-immunometric tests developed here are suitable for this application, as they allow the detection of bacteria grown not only at the body temperature (37°C), but also at lower temperatures (20°C and 28°C). It was also shown in a previous study that anti-PLA mAbs used in immunoblotting or dot-ELISA detected *Y. pestis* strains grown at 28°C, although lower temperatures were not tested [19]. In the present work, the PLA-dipstick was able to detect *Y. pestis* whatever its growth temperature, with a sensitivity of ≈10^6^ cfu/well, while the PLA-EIA reached a sensitivity of 10^4^ cfu/well for bacteria grown at 20°C, and even of 10^3^ cfu/well for *Y. pestis* grown at 28°C. Two previous works that used anti-PLA mAbs for *Y. pestis* detection did not determine the sensitivity of the immunoblotting and dot-ELISA tests used [19], [51]. The high sensitivity of our PLA-EIA therefore offers the possibility to determine the infectious status of fleas because the mean amount of *Y. pestis* present in a single infected flea found on rodent’s carcasses in burrows has been shown to range between 0.6×10^5^ and 0.4×10^6^cfu [66].

In conclusion, this study is the first description of a very sensitive PLA-EIA assay and of a rapid PLA-dipstick test suitable for detection of *Y. pestis* in fleas and environmental samples. Since the PLA-dipstick is less sensitive than the PLA-EIA, the dipstick would be useful as a first line test in the field, and when a higher sensitivity is required, it could be completed with the PLA-EIA as a second line rapid test performed under laboratory conditions. Furthermore, since the PLA-dipstick is F1-independent, it would represent a valuable alternative test if a naturally or genetically engineered F1-negative *Y. pestis* is present or released in the environment.

In conclusion, we have developed two *Y. pestis*-specific immunometric tests that could be valuable complements to the F1-dipstick for the rapid diagnosis of human plague cases, and that have the additional advantage of being usable for the easy and fast detection of the plague agent in environmental samples and in fleas.

## Supporting Information

Figure S1 **SDS-PAGE and Coomassie blue staining of recombinant PLA. (A)** Purity of PLA isolated from inclusion bodies. SDS PAGE in a 10–15% gel. M: molecular weight markers in kDa; 1: total proteins from inclusion bodies; 2: purified recombinant PLA after passage through an Ni-NTA column. **(B)** Refolding of PLA. Recombinant refolded PLA was migrated in 13% SDS PAGE after incubation at room temperature (RT) or denatured at 95°C in Laemmli buffer containing 0.1% SDS. M: Molecular weight markers in kDa.
(TIF)

Figure S2 **Hybridoma selection by ELISA.** *E. coli* BL21(*pla*) (dark blue) and BL21 wild type (negative control, pale blue) were immobilized in 96-well microtiter plates. Hybridoma culture supernatants were incubated with the immobilized bacteria and AChE labeled goat anti-mouse IgG+M antibodies were used as tracers. Non-specific binding (NSB) of the tracer antibodies is shown. The asterisks (*) indicate some clones that were selected for their good specificity.
(TIF)

Table S1 **Bacterial strains and plasmids used in this study.**
(DOCX)

Table S2 **Examples of results of the combinatorial analysis of anti-PLA mAbs.** Each pair of antibodies was analyzed in a two-site immunometric test. One antibody was immobilized on solid phase (capture antibody) and tested in combination with another biotin-labeled antibody (tracer antibody), using BL21(*pla*) as antigen. Empty boxes: AU_414_<0.1; **+**: 0.1<AU_414_<0.2; **++**: 0.2<AU_414_<0.4; **+++**: AU_414_>0.4.
(DOC)
