# Supplementary figures and images for: Fast and Simple Detection of Yersinia pestis Applicable to Field Investigation of Plague Foci
Source: PLoS One. 2013 Jan 29;8(1):e54947. doi: 10.1371/journal.pone.0054947 (PMC3558477; doi:10.1371/journal.pone.0054947)

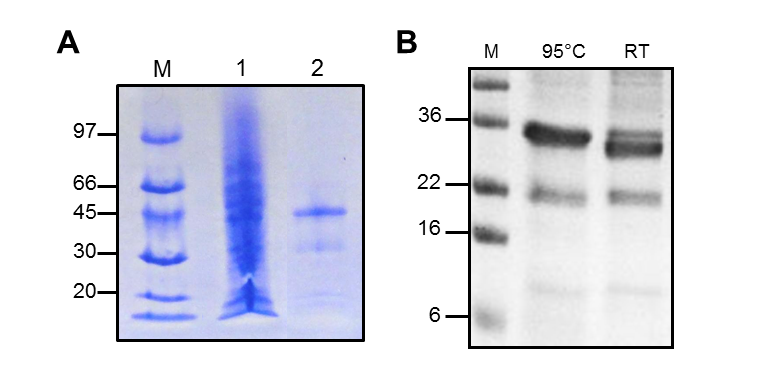

Supplement: Figure S1 — SDS-PAGE and Coomassie blue staining of recombinant PLA. (A) Purity of PLA isolated from inclusion bodies. SDS PAGE in a 10–15% gel. M: molecular weight markers in kDa; 1: total proteins from inclusion bodies; 2: purified recombinant PLA after passage through an Ni-NTA column. (B) Refolding of PLA. Recombinant refolded PLA was migrated in 13% SDS PAGE after incubation at room temperature (RT) or denatured at 95°C in Laemmli buffer containing 0.1% SDS. M: Molecular weight markers in kDa. (TIF) [file pone.0054947.s001.tif]

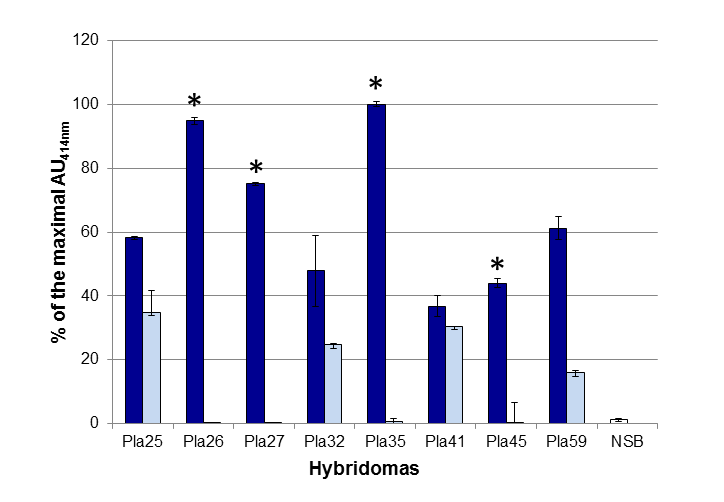

Supplement: Figure S2 — Hybridoma selection by ELISA. E. coli BL21(pla) (dark blue) and BL21 wild type (negative control, pale blue) were immobilized in 96-well microtiter plates. Hybridoma culture supernatants were incubated with the immobilized bacteria and AChE labeled goat anti-mouse IgG+M antibodies were used as tracers. Non-specific binding (NSB) of the tracer antibodies is shown. The asterisks (*) indicate some clones that were selected for their good specificity. (TIF) [file pone.0054947.s002.tif]
