# Supplementary material for: Fast and Simple Detection of Yersinia pestis Applicable to Field Investigation of Plague Foci
Source: PLoS One. 2013 Jan 29;8(1):e54947. doi: 10.1371/journal.pone.0054947 (PMC3558477; doi:10.1371/journal.pone.0054947)
Supplement: Table S1 — Bacterial strains and plasmids used in this study. (DOCX) [file pone.0054947.s003.docx]

| **STRAINS** | **Relevant characteristics** | **Source** |
| --- | --- | --- |
| ***Yersinia pestis*** |  |  |
| CO92 | Wild type, biovar Orientalis | [1] |
| IP882 | Wild type, biovar Orientalis | Yersinia Research Unit, IP |
| IP542 | Wild type, biovar Antiqua | Yersinia Research Unit, IP |
| IP611 | Wild type, biovar Antiqua | Yersinia Research Unit, IP |
| IP516 | Wild type, biovar Medievalis | Yersinia Research Unit, IP |
| 1865 | Wild type, biovar Medievalis | Yersinia Research Unit, IP |
| 6/69∆pPla | 6/69 cured of pPla, biovar Orientalis | This study |
| CO92∆*caf* | CO92 deleted of the *caf* operon | [2] |
| ***Yersinia pseudotuberculosis*** |  |  |
| IP32953 | Wild type, serotype I | [3] |
| IP31629 | Wild type, serotype I | Yersinia Research Unit, IP |
| IP32680 | Wild type, serotype II | [4] |
| IP33377 | Wild type, serotype III | Yersinia Research Unit, IP |
| IP33434 | Wild type, serotype III | Yersinia Research Unit, IP |
| IP31833 | Wild type, serotype IV | Yersinia Research Unit, IP |
| IP32843 | Wild type, serotype V | Yersinia Research Unit, IP |
| 487/90 | Wild type, serotype VI | M. Simonet, IP Lille |
| ***Escherichia*** ***coli*** |  |  |
| BL21 | F- *ompT hsdS*_B_ (r_B_-m_B_-) *gal dcm* | Invitrogen |
| BL21(*pla*) | BL21 harboring pET22b-*pla* | This study |
| ***Erwinia pyrifoliae*** |  |  |
| CIP106111 | Wild type | Collection IP |
| ***Salmonella enterica*** |  |  |
| CIP104474 | Serovar Typhimurium | Collection IP |
| **PLASMIDS** | **Characteristics** | **Source** |
| pET22b(+) | Amp^R^, pBR322 origin, *lacI*, N-terminal PelB leader with signal peptidase site, C-terminal His tag | Novagen |
| pET22b-*pla* | *pla* cloned into the *Nde*I and *Xho*I sites of pET22b(+) | This study |

IP: Institut Pasteur

1. Parkhill J, Wren BW, Thomson NR, Titball RW, Holden MTG, et al. (2001) Genome sequence of *Yersinia pestis*, the causative agent of plague. Nature 413: 523-527.

2. Derbise A, Cerdà Marín A, Ave P, Blisnick T, Huerre M, et al. (2012) An encapsulated *Yersinia pseudotuberculosis* is a highly efficient vaccine against pneumonic plague. PLoS Negl Trop Dis 6: e1528.

3. Chain PS, Carniel E, Larimer FW, Lamerdin J, Stoutland PO, et al. (2004) Insights into the evolution of *Yersinia pestis* through whole-genome comparison with *Yersinia pseudotuberculosis*. Proc Natl Acad Sci USA 101: 13826-13831.

4. Blisnick T, Ave P, Huerre M, Carniel E, Demeure CE (2008) Oral vaccination against bubonic plague using a live avirulent *Yersinia pseudotuberculosis* strain. Infect Immun 76: 3808-3816.
