# Supplementary material for: Fast and Simple Detection of Yersinia pestis Applicable to Field Investigation of Plague Foci
Source: PLoS One. 2013 Jan 29;8(1):e54947. doi: 10.1371/journal.pone.0054947 (PMC3558477; doi:10.1371/journal.pone.0054947)
Supplement: Table S2 — Examples of results of the combinatorial analysis of anti-PLA mAbs. Each pair of antibodies was analyzed in a two-site immunometric test. One antibody was immobilized on solid phase (capture antibody) and tested in combination with another biotin-labeled antibody (tracer antibody), using BL21(pla) as antigen. Empty boxes: AU414<0.1; +: 0.1<AU414<0.2; ++: 0.2<AU414<0.4; +++: AU414>0.4. (DOC) [file pone.0054947.s004.doc]

|  |  | **Tracer antibody** | | | | | | | | | | | | | | | | | |
| --- | --- | --- | --- | --- | --- | --- | --- | --- | --- | --- | --- | --- | --- | --- | --- | --- | --- | --- | --- |
|  |  | **Pla1** | **Pla7** | **Pla8** | **Pla17** | **Pla18** | **Pla21** | **Pla26** | **Pla27** | **Pla30** | **Pla33** | **Pla35** | **Pla36** | **Pla42** | **Pla43** | **Pla44** | **Pla45** | **Pla47** | **Pla50** |
| **Capture antibody** | **Pla7** | **++** | **+** | **+** | **+** | **+** | **++** | **++** |  |  | **+** | **+++** | **++** | **+** |  |  | **+** |  | **+** |
|  | **Pla8** |  |  |  |  |  |  |  |  |  |  | **+** |  |  |  |  |  |  |  |
|  | **Pla17** |  |  | **+** |  |  |  |  |  |  |  | **+** |  |  |  |  |  |  |  |
|  | **Pla18** |  |  |  |  |  |  |  |  |  |  | **+** |  |  |  |  |  |  |  |
|  | **Pla21** | **+** |  |  | **++** |  | **+** | **+** |  |  |  | **+** | **+** | **+** |  |  |  |  |  |
|  | **Pla26** | **+** |  |  | **+** |  |  |  |  |  |  | **+** | **+** | **+** |  |  |  |  |  |
|  | **Pla27** |  |  |  |  |  | **++** | **++** |  |  | **+** | **++** | **++** | **+** |  |  |  |  |  |
|  | **Pla28** |  |  |  | **+** |  | **+** | **+** |  |  | **+** | **++** | **++** | **+** |  |  |  |  |  |
|  | **Pla30** |  |  |  |  |  |  |  |  |  |  | **+** |  |  |  |  |  |  |  |
|  | **Pla33** | **++** |  | **+** | **+++** |  | **++** | **++** |  | **+** | **+** | **+++** | **++** | **+** | **+** | **++** | **++** | **+** | **+** |
|  | **Pla35** | **+** |  | **+** | **+++** |  | **+** | **+** |  |  |  | **++** |  | **+** | **+** | **+** |  |  |  |
|  | **Pla36** | **++** |  | **+** | **+++** | **+** | **++** | **++** | **+** | **+** | **++** | **++** | **++** | **+** | **+** | **+** |  |  |  |
|  | **Pla41** | **+** |  |  |  |  |  |  |  |  |  |  |  |  |  |  |  |  |  |
|  | **Pla42** | **+** |  |  | **++** |  | **+** | **+** |  |  |  | **++** | **+** | **+** |  |  |  |  |  |
|  | **Pla43** | **+** |  |  | **++** |  | **+** |  |  | **+** |  | **+** | **+** |  |  | **+** |  |  |  |
|  | **Pla44** |  |  |  |  |  |  |  |  |  |  | **++** | **+** | **+** | **+** |  |  |  |  |
|  | **Pla45** |  |  | **+** | **+** |  | **++** | **++** |  |  | **++** | **+++** | **+++** | **++** |  |  |  | **+** |  |
|  | **Pla46** |  |  |  |  |  |  |  |  |  |  | **+** |  |  |  |  |  |  |  |
|  | **Pla49** |  |  |  |  |  |  |  |  |  |  | **+** |  |  |  |  |  |  |  |
|  | **Pla53** |  |  |  | **++** |  | **+** | **+** |  |  |  | **+** | **+** |  |  |  |  |  |  |
